# Incorporating structural abnormalities in equivalent dipole layer based ECG simulations

**DOI:** 10.3389/fphys.2022.1089343

**Published:** 2022-12-22

**Authors:** Machteld J Boonstra, Thom F Oostendorp, Rob W Roudijk, Manon Kloosterman, Folkert W Asselbergs, Peter Loh, Peter M Van Dam

**Affiliations:** ^1^ Department of Cardiology, Division Heart and Lungs, University Medical Center Utrecht, Utrecht University, Utrecht, Netherlands; ^2^ Radboud University Nijmegen Medical Centre, Donders Institute for Brain, Cognition and Behavior, Nijmegen, Netherlands; ^3^ Faculty of Population Health Sciences, Institute of Cardiovascular Science, University College London, London, United Kingdom; ^4^ Health Data Research UK and Institute of Health Informatics, University College London, London, United Kingdom; ^5^ ECG Excellence BV, Nieuwerbrug aan den Rijn, Weijland, Netherlands

**Keywords:** electrocardiogram (ECG), equivalent dipole layer, ECGsim, myocardial disease, cardiac activation, simulation

## Abstract

**Introduction:** Electrical activity of the myocardium is recorded with the 12-lead ECG. ECG simulations can improve our understanding of the relation between abnormal ventricular activation in diseased myocardium and body surface potentials (BSP). However, in equivalent dipole layer (EDL)-based ECG simulations, the presence of diseased myocardium breaks the equivalence of the dipole layer. To simulate diseased myocardium, patches with altered electrophysiological characteristics were incorporated within the model. The relation between diseased myocardium and corresponding BSP was investigated in a simulation study.

**Methods:** Activation sequences in normal and diseased myocardium were simulated and corresponding 64-lead BSP were computed in four models with distinct patch locations. QRS-complexes were compared using correlation coefficient (CC). The effect of different types of patch activation was assessed. Of one patient, simulated electrograms were compared to electrograms recorded during invasive electro-anatomical mapping.

**Results:** Hundred-fifty-three abnormal activation sequences were simulated. Median QRS-CC of delayed versus dyssynchronous were significantly different (1.00 vs. 0.97, *p* < 0.001). Depending on the location of the patch, BSP leads were affected differently. Within diseased regions, fragmentation, low bipolar voltages and late potentials were observed in both recorded and simulated electrograms.

**Discussion:** A novel method to simulate cardiomyopathy in EDL-based ECG simulations was established and evaluated. The new patch-based approach created a realistic relation between ECG waveforms and underlying activation sequences. Findings in the simulated cases were in agreement with clinical observations. With this method, our understanding of disease progression in cardiomyopathies may be further improved and used in advanced inverse ECG procedures.

## 1 Introduction

The ECG provides valuable insight into the electrical activity of the heart (Kligfield et al., 2007). In clinical practice, interpretation of changes in the ECG due to pathology are mainly based on ECG-based pattern recognition (Rautaharju et al., 1998; Hancock et al., 2009; Surawicz et al., 2009). General understanding of the effect of abnormal electrical activity in diseased myocardium on intracardiac and body surface potentials (BSP) is obtained with invasive electro-anatomical mapping (EAM) studies and ECG simulation studies. Through ECG simulation, better understanding of the electrophysiological behavior of myocardial substrate can be achieved (Dössel et al., 2021). For example, the effect of different types and locations of diseased myocardium on simulated BSP may provide valuable clinical information about disease onset and/or progression.

However, the relation between abnormal ventricular activation and the corresponding ECGs still requires more fundamental understanding. Specifically on the relation between ECG waveform changes and the pathological wave propagation in the presence of structural myocardial disease. Invasive mapping procedures have already provided a lot of information about local electrical dysfunction of the myocardium. The presence of late potentials, low bipolar and unipolar voltages and fractionation of local electrograms is directly related to the disruption of the activation sequence due to the presence of fibrous or fibrofatty tissue (De Bakker et al., 1993; Aliot et al., 2009). Additionally, the voltage of unipolar signals is shown to be related to the presence of epicardial and/or transmural scar tissue (Marchlinski et al., 2000; Soejima et al., 2002; Glashan et al., 2018).

The relation between abnormal ventricular activation and ECG waveforms can also be investigated by ECG simulation. To simulate the ECG, two types of models are required. The first model is the cardiac source model, representing the electrical currents generated by the myocardial cells. And second, the volume conductor model, which describes the effect of these generated currents on potentials throughout the torso. A well-known interactive ECG simulation program is *ECGsim* (van Dam et al., 2010; van Oosterom et al., 2011); a program based on the equivalent dipole layer (EDL) cardiac source model and boundary element method-based volume conductor. The EDL source model is based on work by Wilson et al. (1933); Geselowitz (1963) and shown to be also valid in homogeneous anisotropic tissue by Wilson et al. (1933); Geselowitz (1963). He observed that potentials generated outside the heart by the electric activity of all myocardial cells is proportional to the potentials generated by a simulated dipole layer at the surface of that mass, with the dipole layer strength proportional to the upstroke of the local transmembrane potential provided we can assume homogeneous anisotropy ratios. The EDL is positioned at the endocardial and epicardial surface bounding the myocardium. At each element of this surface model, local source strength is defined by the local transmembrane potential. Adjusting the local timing of depolarization or repolarization result in changes in the simulated BSP.

The presence of diseased myocardium, such as scar, within the myocardial mass, breaks the equivalence of the dipole layer. In order to restore the equivalence, the boundary between normal and diseased myocardium must be included in the model. In earlier studies, old myocardial infarctions were simulated by removing parts of the ventricular anatomical model, thereby creating a hole in the anatomy (Oostendorp and Ham, 2001). This method can be used for cases of homogeneous transmural scar without any surviving myocardium, as is often the case in ischemic heart disease. However, in other cases of ischemic heart disease, surviving tissue is present within dense scarred regions. Furthermore, in case of progressive fibrofatty myocardial scarring, as in some inherited cardiomyopathies, strands of normal and fibrofatty myocardium are intermingled in diseased areas. Thus, diseased regions remain partially electrically active which consequently affects recorded potentials. The aim of this study was to evaluate a new method to incorporate local electrical abnormalities into the EDL simulation method. In this simulation study, we studied the effect on corresponding simulated BSP. Additionally, we compared simulations with our method to recordings in one patient who underwent an invasive EAM.

## 2 Materials and methods

### 2.1 EDL-based simulation of potentials

**FIGURE 1:**
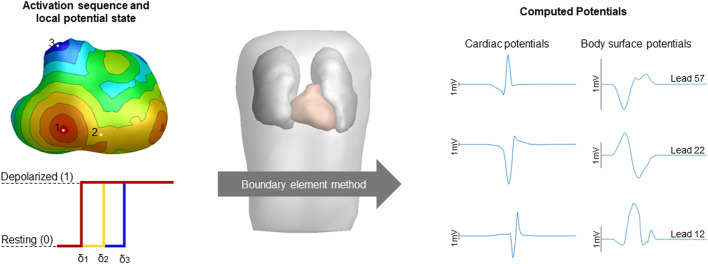
Schematic overview of the computation of potentials. The simulated activation sequences, constructed transmembrane potentials and the boundary element method compute the volume conductor effect to compute cardiac and body surface potentials. Activation timing is displayed from red (early) to blue (late), corresponding local potential states were displayed for three sites of interest with the color of the displayed potential corresponding to the local activation timing with δ denoting local activation timing. Computed cardiac and body surface potentials were displayed with the height of the black bar indicating 1 mV. The displayed body surface potentials are taken from the 64-lead setup, specifically lead 12 positioned two intercostal spaces above V1, lead 22 positioned at the lower sternum and lead 57 positioned at the right side of the back are displayed.

EDL-based ECG simulation relates the electrical activity at the endocardial and epicardial surface to potentials within and at the body surface (Figure 1). In the EDL-source description, the potential ( $\Phi \left(t,\vec{y}\right)$ ) generated at any location $\vec{y}$ and time t on and within the body surface is given by:

$$\Phi \left(t,\vec{y}\right)=\int_{S_{v}}A\left(\vec{y},\vec{x}\right)V_{m}\left(t,\vec{x}\right)d\omega \left(\vec{y},\vec{x}\right)$$

With $V_{m}\left(t,\vec{x}\right)$ the upstroke of the transmembrane potential at position $\vec{x}$ on the surface of the ventricular myocardial mass ( $S_{v}$ ) at time t , $d\omega (\vec{y},\vec{x}$ ) the solid angle of the infinitesimal surface element $dS\left(\vec{x}\right)$ observed from $\vec{y}$ . $A\left(\vec{y},\vec{x}\right)$ denotes the transfer matrix that expresses the effect of the volume conductor. A model of the volume conductor is typically obtained from MR or CT imaging. The surfaces of all anatomical structures are discretized as closed triangulated surface meshes. $A\left(\vec{y},\vec{x}\right)$ is computed using the boundary element method from each triangle of the discretized ventricular surface towards each observation point at or within the torso (Oostendorp and van Oosterom, 1991; Tate et al., 2019)

The electric activity at the myocardial surface at position $\vec{x}$ follows the local transmembrane potential waveform (van Oosterom, 2002; van Oosterom, 2003). At each node, the source strength is proportional to the local transmembrane potentials. Based on the observation that the potential step across the activation wavefront within the myocardium is 40 mV, the dipole layer strength is calibrated such that a completely activated equivalent source element generates the same potential drop (van Oosterom et al., 1989; van Oosterom, 2001). The local potential step across a uniform dipole layer is equal to the dipole layer strength (in A/m) divided by the conductivity value of the heart (in S/m) (Plonsey and Barr, 2011). With a potential step of 40 mV and conductivity value of 0.2 S/m, the normal dipole layer strength is 8 μA/mm.

In the research described in this paper, we specifically studied the effect of activation wave changes on simulated BSP, thus focusing on the depolarization phase. The local potential $V_{m}(t;\delta$ ) is defined as a step function where the local potential ( $V_{m}$ ) in the depolarized state was set to one and in the resting state to zero. The local body surface potential is described as a function of the local depolarization ( δ ) time at the surface is given by:

$$\Phi \left(t,\vec{y}\right)=\int_{S_{v}}A\left(\vec{y},\vec{x}\right)V_{m}\left(t;\delta \right)d\omega \left(\vec{y},\vec{x}\right)$$

To reflect the propagation of depolarization over each discretized triangle elements at the surface of the myocardium, the source strength at time t of a triangle partially activated is weighted with the fraction of the triangle, that is, activated at t , as previously described (Tate et al., 2019).

#### 2.1.1 Simulating diseased ventricular myocardium: The patch

**FIGURE 2:**
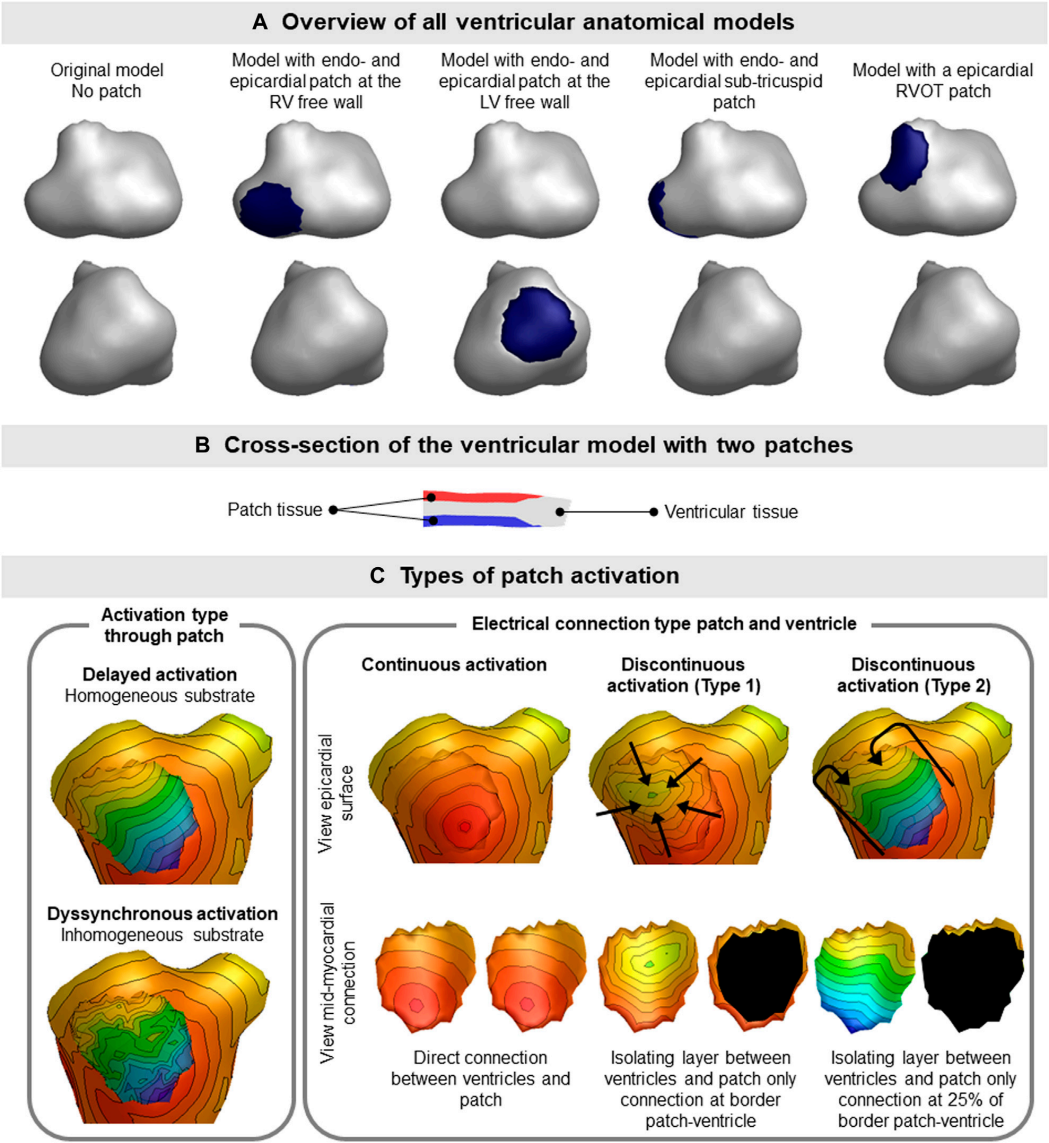
Ventricular patch model overview and simulation characteristics. **(A)** The four different patch locations used for the simulation studies are displayed. The location of the patch is indicated by the blue region on the epicardium. Where indicated with endo- and epicardial patch, an opposing endocardial is present with the same size as the displayed epicardial patch. **(B)** Patches (red, endocardial and blue, epicardial) were embedded within the ventricular tissue (gray), thereby not changing the outer shape of the ventricular model. **(C)** Different types of activation sequences (delayed, dyssynchronous, continuous and discontinuous) of the patches are displayed as local activation timing maps from red (early) to blue (late) with isochrone steps of 10 ms. The isolating layer between the ventricles and patch is indicated in black on the inside of the patch. The ventricular activation sequence of the myocardium (excluding the patch) used in this study was equal for all models.

Because the presence of fibrofatty tissue breaks the equivalence of the dipole layer at the myocardial surface, we divided our segmented ventricular model into separate parts wherein we either simulate normal or diseased myocardium. By specifically including these as separate components within the segmented ventricular model, the equivalence of the EDL is restored (Figure 2A). This provides the opportunity to represent both electrically active and passive myocardial cells within the specific region, similarly to a fibroblast model. The parts representing diseased, non-activatable myocardium are hereafter called patches. Different activation characteristics were assigned to patches to represent different types of diseased myocardium. The patches were embedded within the segmented ventricular model so that the outer shape of the ventricular model remains intact to a model without patches, but creating a mid-myocardial border between normal and diseased parts. At the location of the patch, the nodes of the normal ventricular model are pushed inward, creating a local dent (Figure 2B). The inner surface of the patch is an exact copy of the nodes at the patch-ventricular border so the nodes were exactly opposing each other. Separate volume conductor models were computed per source [*e.g.,* ventricles and/or patch (es)].

Cardiac CT was used to create patient specific anatomical models of the ventricular myocardium, blood pools, lungs and torso in the current study. The assigned conductivity values were 0.2 S/m for the thorax, patch and ventricular myocardium, 0.04 S/m for the lungs and 0.6 S/m for the blood cavities. (Gulrajani and Mailloux, 1983; Huiskamp and Van Oosterom, 1988; van Oosterom and Huiskamp, 1989; van Oosterom, 2001). To generate the local potential, we set the local potential to 0 (resting) or 1 (depolarized) depending on the local depolarization timing (Figure 1). The contribution of currents generated by each part (healthy and diseased) on BSP was accounted for based on the superposition principle.

### 2.2 Simulation study

To assess the effect of different types of patch activation sequences on BSP, one patient specific (male, 57 y.o.) anatomical model was created using GeomPEACS (van Dam et al., 2015). The anatomical model contained the triangulated surface meshes of the ventricular myocardium, blood pools, torso and lungs. Using this set, three subsets of ventricular models with endocardial and opposing epicardial patches at different locations in both the right and left ventricle and one model with only an epicardial patch in the right ventricular outflow tract (RVOT) were created (Figure 2A).

#### 2.2.1 Simulating normal ventricular activation

A case of normal ventricular activation (e.g., sinus rhythm) with an intact His-Purkinje network was simulated by using a set of seven distinct foci (four on the left ventricular (LV) and three on the right ventricular (RV) endocardium) as starting points of activation and the fastest route algorithm (van Dam et al., 2009). A constant propagation velocity of 0.85 m/s along the myocardial surface was selected, and a 2.5 times slower propagation perpendicular to the wall (Figure 2C). For all anatomical models, the normal ventricular activation sequence was the same.

#### 2.2.2 Simulating patch activation

**FIGURE 3:**
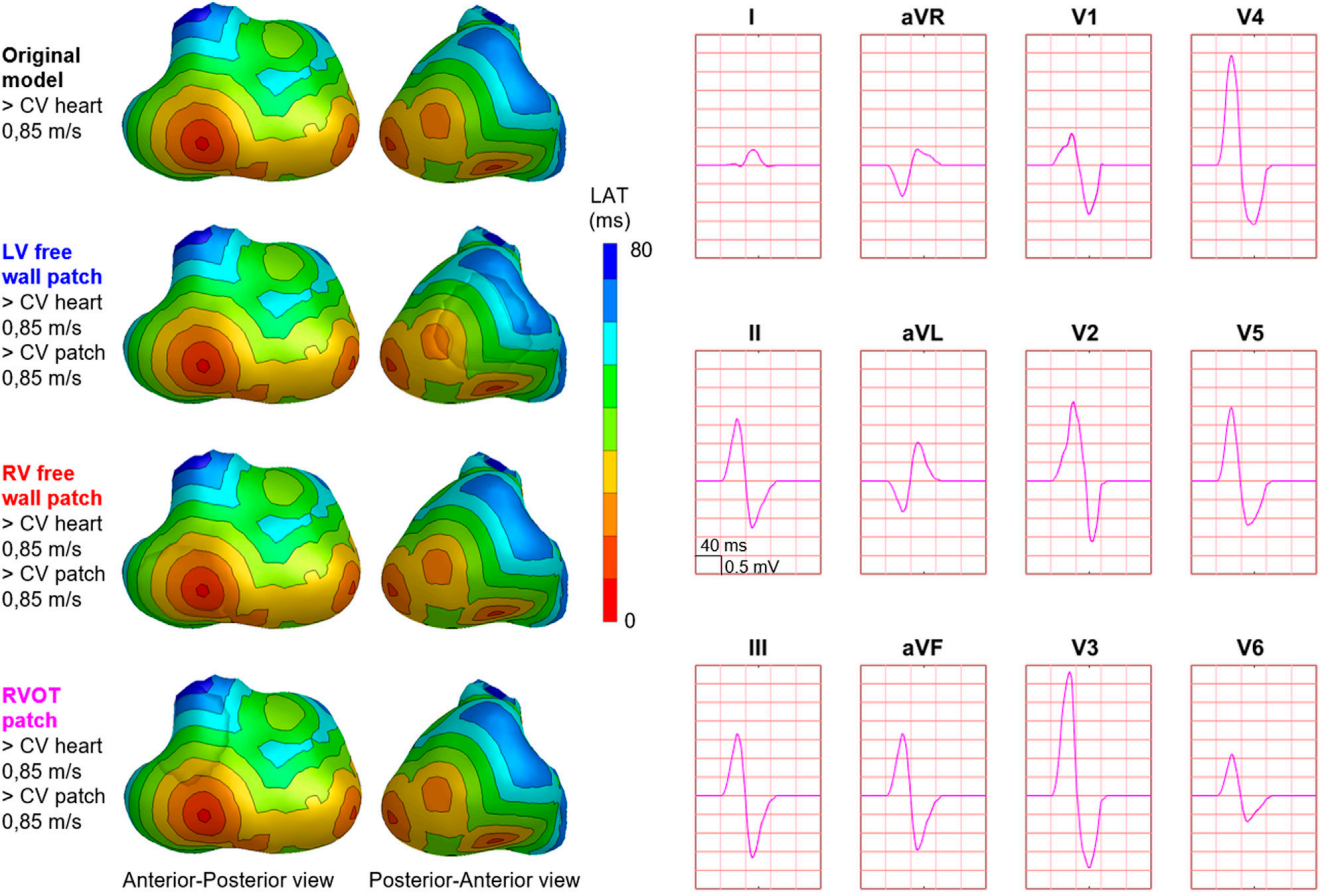
Effect of patch incorporation within the ventricular model. For both the original model and three of the models with patch, normal ventricular and patch activation were computed. Activation sequences were displayed from red (early) to blue (late). All simulated BSP signals overlap, as can be observed in the presented standard 12-leed ECG. One cube in the 12-lead ECG corresponds to 40 ms (width) and 0.5 mV (height) as also indicated in lead II.

The fastest route algorithm was also used to compute the patch activation sequences, with a set propagation velocity and additional characteristics depending on the type of simulated substrate. When using the same propagation velocity for the patch and the ventricles, simulated BSP were the same as the original model (Figure 3), as expected.

Three distinct types of abnormal patch activation sequences were simulated to assess their effect on simulated BSP (Table 1): 1) delayed patch activation due to homogeneous activation wave slowing, 2) dyssynchronous patch activation due to inhomogeneous myocardial substrate and 3) discontinuous patch activation by simulating an isolating layer between normal ventricular and patch myocardium. For the delayed patch simulations, propagation velocity ranged between 0.85 m/s (normal) and 0.25 m/s (severe disease). To simulate dyssynchronous patch activation, a Gaussian noise generator (*rand* function MATLAB) was used with activation times that differed 0–90 ms from normal. Three types of discontinuous patch activations were simulated (Figure 2C): the part of the patch in direct contact with the myocardium was either fully connected with the normal ventricular model, or only the outer ring of the patch was connected to the ventricular model (type 1), or only the latest 25% of the outer ring were connected to the ventricular model (type 2). Depending on the electrical connection between ventricular and patch tissue (Figure 2C), nodes directly in contact with the ventricular model were assigned the same activation timing as the ventricular nodes.

**TABLE 1:** Summary of patch simulation characteristics.

Patch simulation type	Affected patch	Type of patch activation	Patch simulation characteristics	Connection patch/healthy
Delayed patch activation	Endocardial	Homogeneous	0.25–0.65 m/s + 0 ms random	Inner patch surface 100%
	Epicardial			
Delayed type 1 patch activation	Endocardial	Homogeneous	0.25–0.65 m/s + 0 ms random	Outer ring 100%
	Epicardial			
Delayed type 2 patch activation	Endocardial	Homogeneous	0.25–0.65 m/s + 0 ms random	Outer ring 25%
	Epicardial			
Dyssynchronous patch activation	Endocardial	Inhomogeneous	0.45 m/s + 30–90 ms random	Inner patch surface 100%
	Epicardial			
Dyssynchronous type 1 patch activation	Endocardial	Inhomogeneous	0.45 m/s + 30–90 ms random	Outer ring 100%
	Epicardial			
Dyssynchronous type 2 patch activation	Endocardial	Inhomogeneous	0.45 m/s + 30–90 ms random	Outer ring 25%
	Epicardial			

To account for the presence of fibrofatty tissue, we assumed that with increasing amount of fibrofatty tissue, the percentage of electrically active cells within the region decreases linearly. At some distance, this corresponds to an activation wavefront with a dipole layer strength of less than the normal 8 mA/m. Consequently, the strength of equivalent dipole layer at the surface of the patch was heuristically scaled by the simulated percentage of healthy myocardium within the patch.

#### 2.2.3 Statistical analysis

To compare different simulations to the case without any patch (*i.e.,* assumed as normal), the effect on signal amplitude, QRS duration and QRS morphology was assessed. Furthermore, the most affected leads were determined using a 64-lead BSP simulation setup where the most affected lead was determined as the lead with the lowest Pearson’s correlation coefficient (CC) comparing abnormal to normal QRS. Normally distributed variables were reported as mean ± standard deviation and non-normally distributed data were reported as median with interquartile range. Differences between normally distributed data was tested for significance using unpaired students *t*-test and non-normal data were compared using Mann-Whitney *U* test.

### 2.3 Clinical case - Invasive electro-anatomical mapping

One patient (male, 65 y.o.) referred for invasive EAM and ablation was enrolled. The EAM procedure was clinically indicated because of recurrent ventricular arrhythmia due to structural myocardial disease (arrhythmogenic cardiomyopathy). The patient is a carrier of a pathogenic plakophilin-2 mutation, which is associated with the development of arrhythmogenic cardiomyopathy. As a part of the clinical workup prior to the EAM procedure, the patient underwent cardiac computed tomography imaging. For study purposes, 64-lead BSP mapping was performed on the day prior to EAM. The study protocol was approved by the local institutional review board (UMC Utrecht, Netherlands, protocol nr. 17/628). The patient gave informed consent prior to BSP mapping and the study was conducted according to the declaration of Helsinki. During EAM, the epicardium and RV endocardium were mapped using a cardiac mapping system with the multipolar HD-grid catheter (Advisor™, Ensite Precision, Abbott).

Patient specific anatomical models of the ventricular myocardium, blood pools, lungs and torso were created and electrode positions captured with a 3D camera were registered to the torso model. A patch at the basal region of the RV endocardium was created, in the same region as abnormal electrograms were observed during EAM. Normal ventricular activation with an intact His-Purkinje network was simulated using a set of eight foci in combination with a tuned propagation velocity of 1 m/s. Location of the foci and propagation velocity were tuned to ensure that the simulated QRS-complex waveform in the extremity leads was similar to patient specific recorded QRS-complex waveforms and QRS duration (100 ms). Arrhythmogenic cardiomyopathy is characterized by defects in intercellular connections, e.g., the intercalated discs, resulting in a combination of altered intercellular impulse propagation and progressive fibro-fatty replacement of healthy myocardium. Therefore, dyssynchronous patch activation was simulated using a propagation velocity of 0.85 m/s and adding random noise within the range of 0–50 ms. To represent the presence of fibrofatty tissue, a uniform source strength of 50% (4 mA/m) was used for the patch.

#### 2.3.1 Epicardial and endocardial electrograms

After the procedure, all electrograms obtained prior to ablation were manually checked for validity. The local activation timing was determined at the maximal absolute amplitude of the bipolar signal, which corresponds to the maximum downslope (dV/dt) in unipolar signals. Data were exported as raw electrograms with location, annotated local activation timing and bipolar voltage. For the simulated case, local activation timing was set as the time instance of upstroke of transmembrane potential amplitude. Bipolar electrograms were computed by subtracting the unipolar electrogram of direct neighboring nodes. Per node, bipolar voltage was calculated as the maximum potential difference between the node and any of its neighbors. For both the recorded and the simulated case of the same patient, local activation timing maps and bipolar voltage maps were constructed.

## 3 Results

### 3.1 Simulation study

**FIGURE 4:**
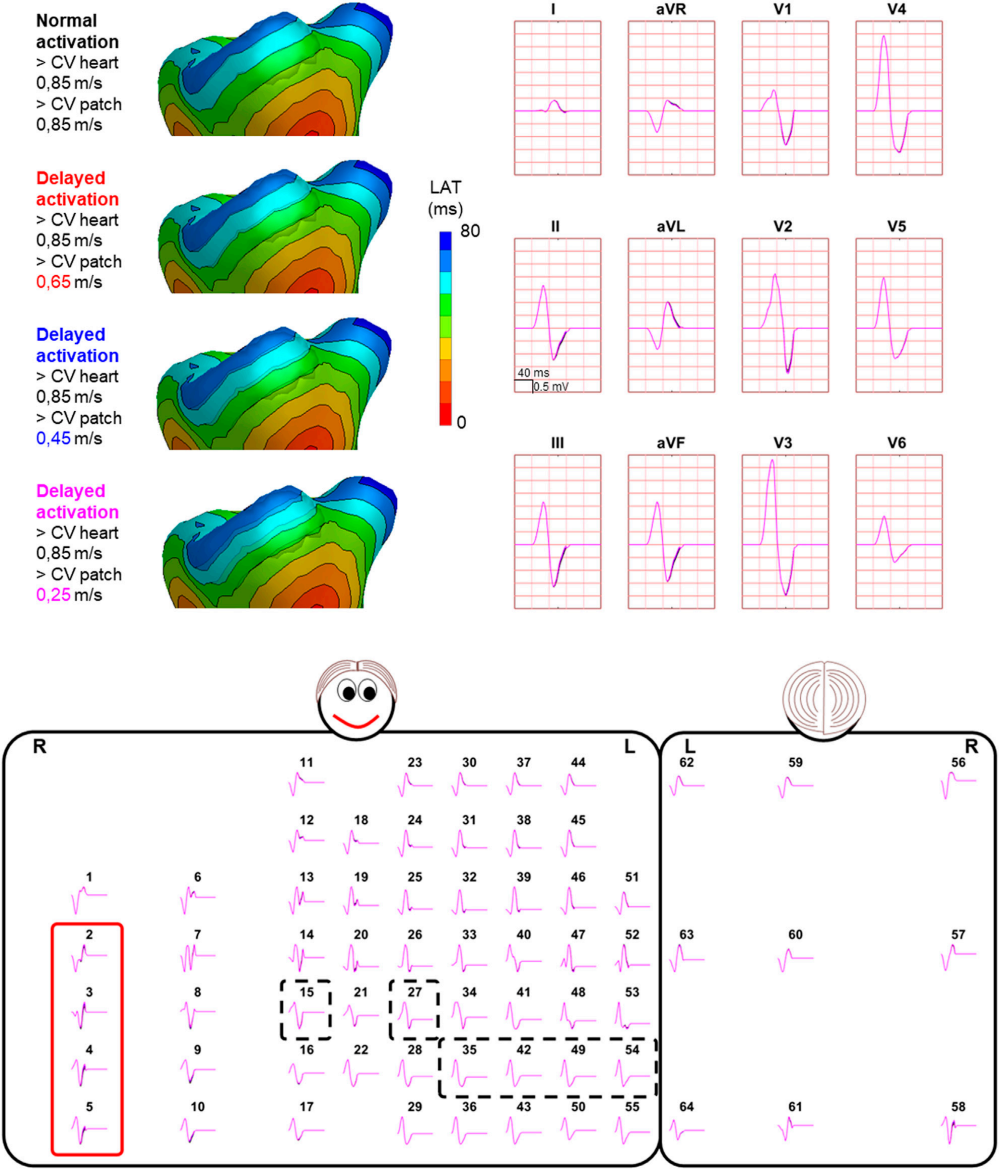
Effect of delayed patch activation on body surface potentials. Representative example of the effect of delayed patch activation reducing the propagation velocity through the patch. Activation sequences of the patches and ventricles are displayed from red (early) to blue (late) with isochrone steps of 10 ms. All simulated BSP are displayed in the simulated 12-lead ECG and the 64-lead BSP. The colors in the ECG correspond to the colors stated in the left column with the simulation characteristics. Within the 64-lead BSP, maximum changes in QRS complex were observed in the leads indicated with the red box. Electrode position of the 12-lead ECG was indicated by the black dashed boxes. One cube in the 12-lead ECG corresponds to 40 ms (width) and 0.5 mV (height) as also indicated in lead II.

**FIGURE 5:**
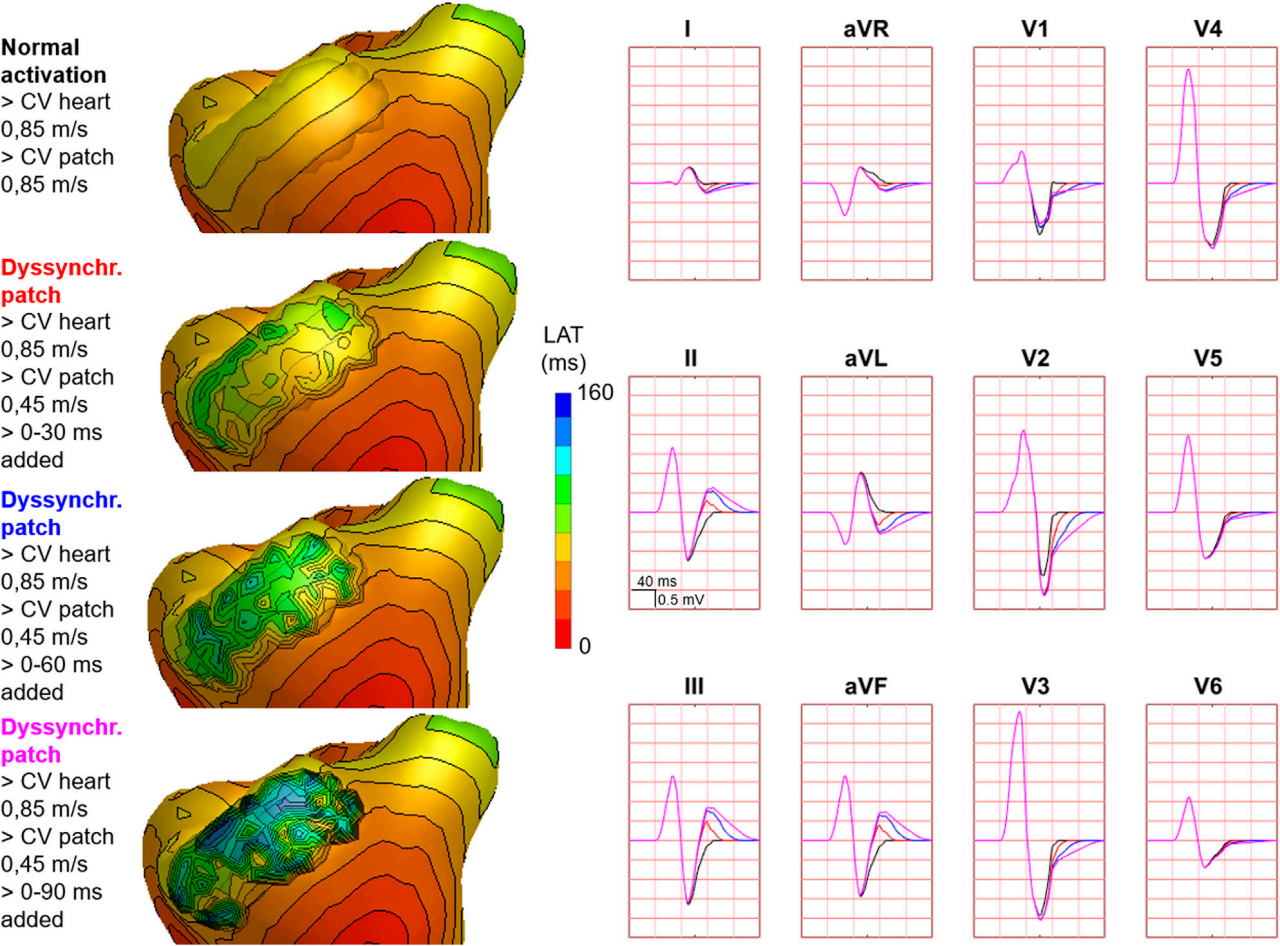
Effect of dyssynchronous patch activation on body surface potentials. Representative example of the effect of dyssynchronous patch activation. Activation sequences of the patches and ventricles are displayed from red (early) to blue (late) with isochrone steps of 10 ms. Simulated BSP are displayed as 12-lead ECG. The colors in the ECG correspond to the colors stated in the left column with the simulation characteristics. Isochronal crowding, that is, observed on the patch increases by increasing the amount of added jitter. One cube in the 12-lead ECG corresponds to 40 ms (width) and 0.5 mV (height) as also indicated in lead II.

**FIGURE 6:**
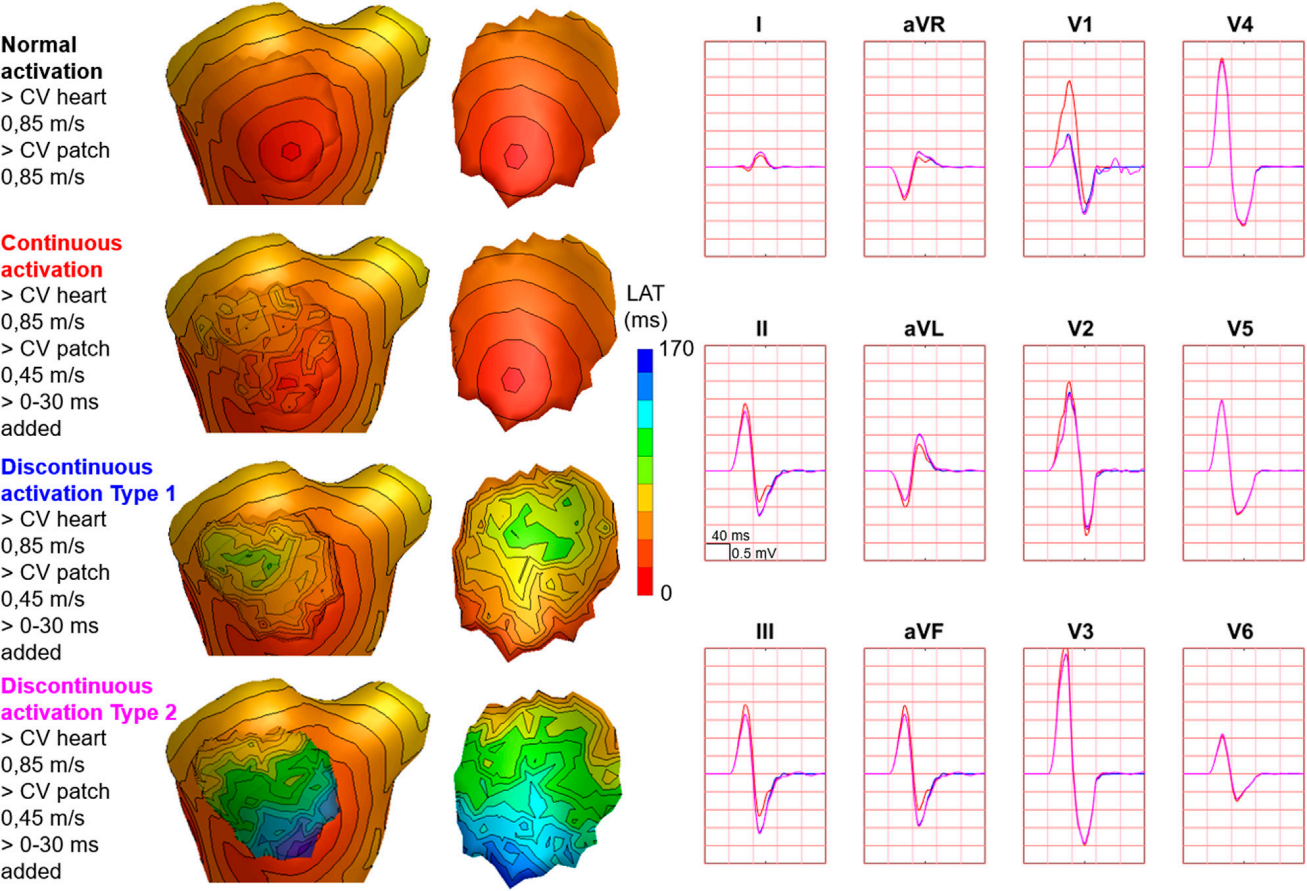
Effect of discontinuous patch activation on body surface potentials. Representative example of the effect of different types of discontinuous patch activations on body surface potentials. Activation sequences of the patches and ventricles are from red (early) to blue (late) with isochrone steps of 10 ms. All simulated BSP are displayed in the simulated 12-lead ECG. The colors in the ECG correspond to the colors stated in the left column with the simulation characteristics. With different patch simulations, most changes were observed in V1, where the magnitude of the effect differed between simulations. One cube in the 12-lead ECG corresponds to 40 ms (width) and 0.5 mV (height) as also indicated in lead II.

**FIGURE 7:**
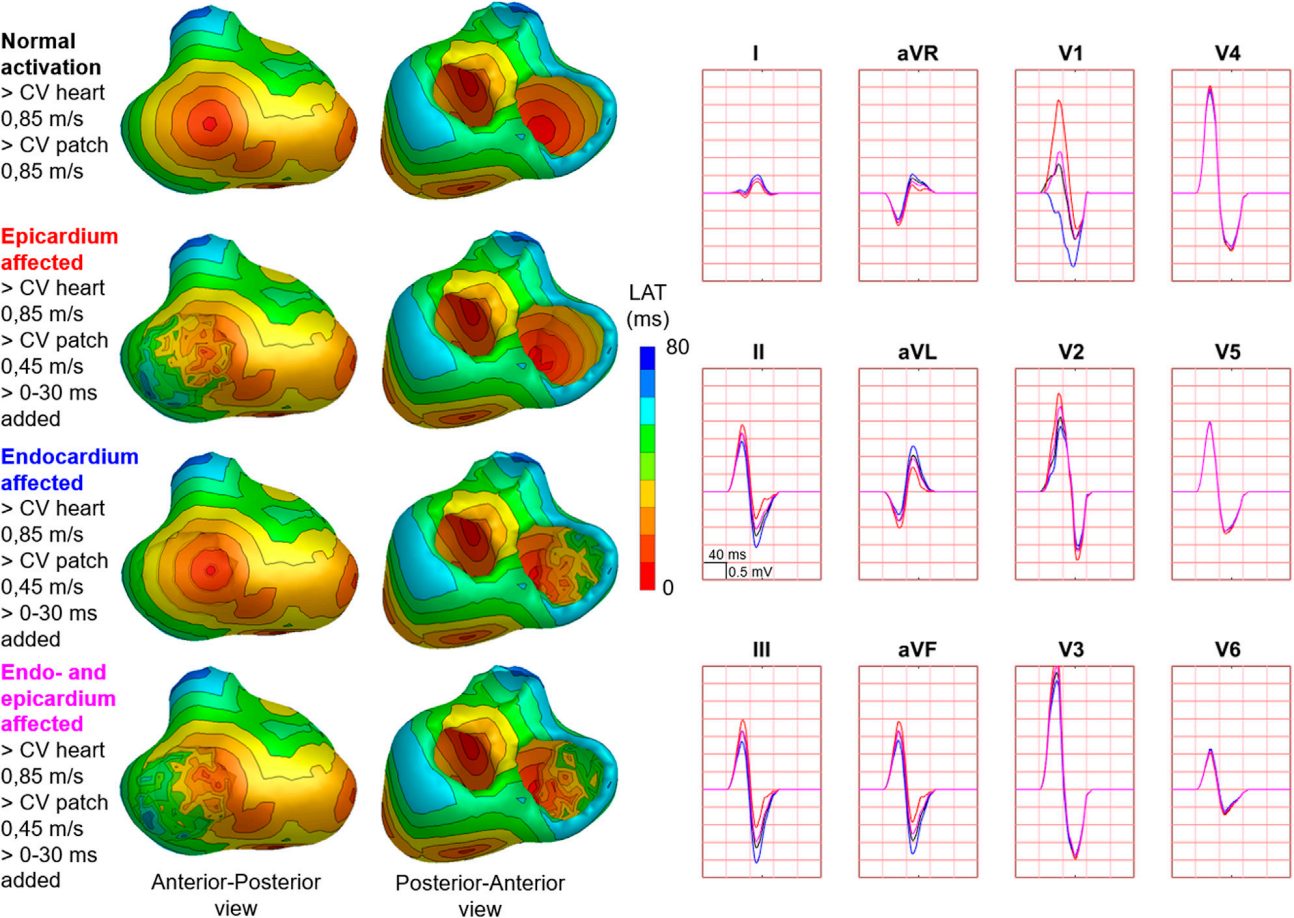
Effect of endocardial vs. epicardial disease on body surface potentials. Representative example of the effect of diseased endocardial and/or epicardial patch activation on body surface potentials. Activation sequences of the patches and ventricles are displayed from red (early) to blue (late) with isochrone steps of 10 ms. All simulated BSP are displayed in the simulated 12-lead ECG. The colors in the ECG correspond to the colors stated in the left column with the simulation characteristics. With endocardial vs. epicardial patch location, the effect in leads with maximum effect was opposed. One cube in the 12-lead ECG corresponds to 40 ms (width) and 0.5 mV (height) as also indicated in lead II.

**FIGURE 8:**
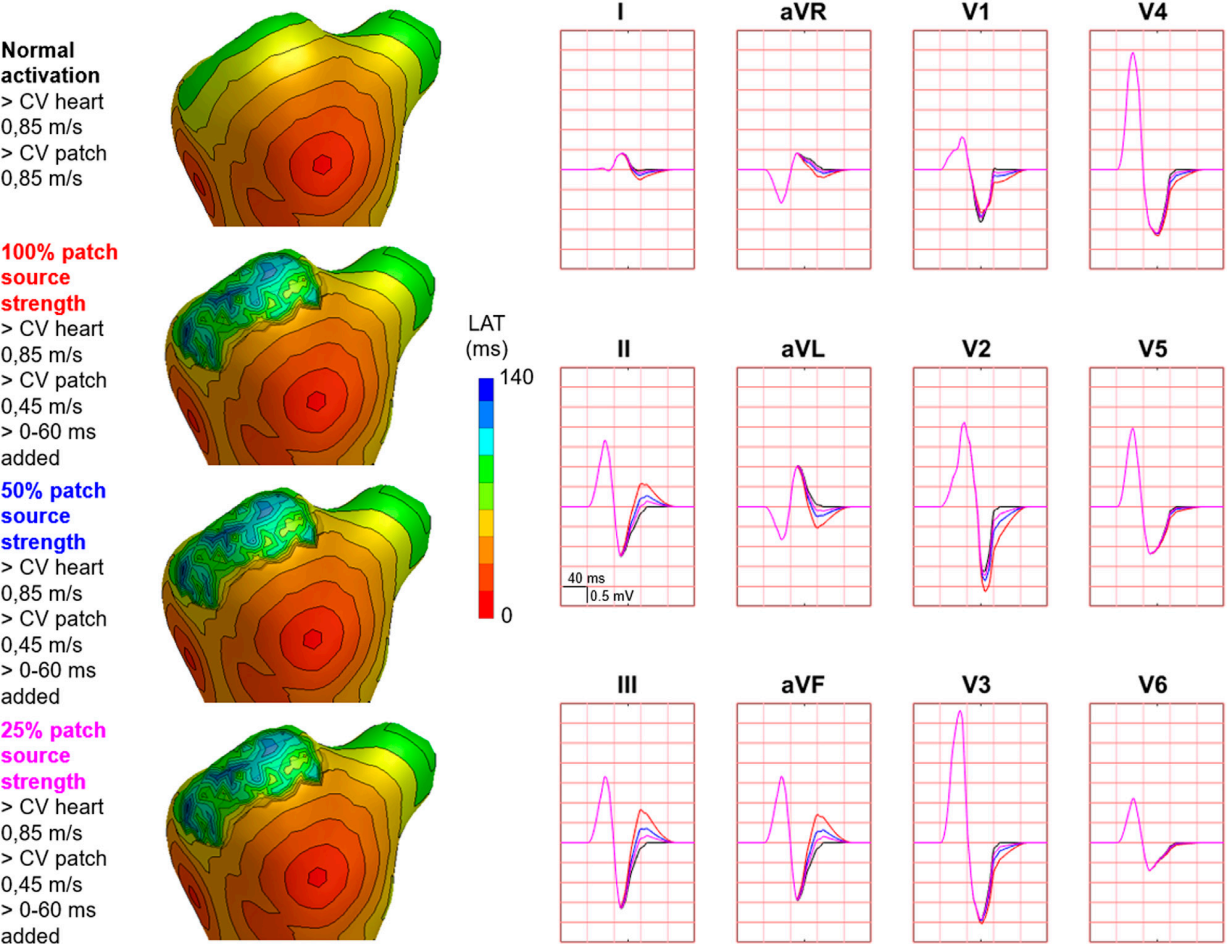
Effect of decreasing source strength on body surface potentials. Representative example of the effect of source strength reduction body surface potentials. Activation sequences of the patches and ventricles are displayed from red (early) to blue (late) with isochrone steps of 10 ms. All simulated BSP are displayed in the simulated 12-lead ECG. The colors in the ECG correspond to the colors stated in the left column with the simulation characteristics. With decreasing source strength, the magnitude of effect on the BSP reduced. One cube in the 12-lead ECG corresponds to 40 ms (width) and 0.5 mV (height).

**FIGURE 9:**
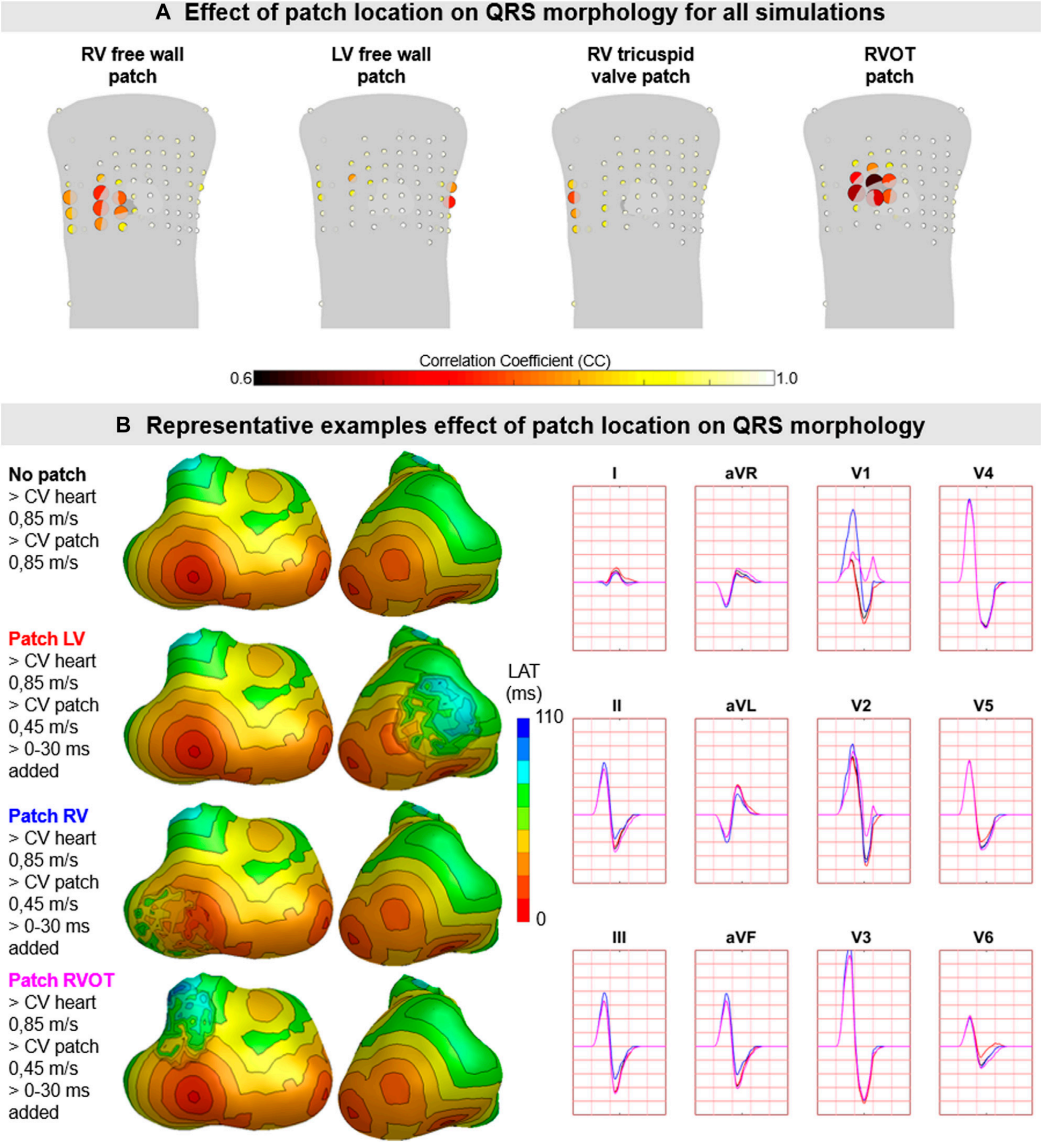
The effect of patch location on simulated body surface potentials. **(A)** The mean ± standard deviation of correlation coefficient (CC) per BPS lead between normal and diseased BSP. Each lead is represented by a dot with the color representing average CC (range 0.6–1) and size representing the magnitude in standard deviation of CC. The heart contour (light grey) with patch (dark grey) is displayed within the torso. **(B)** Representative example of the effect of patch location on BSP. Activation sequences of the patches and ventricles are displayed from red (early) to blue (late) with isochrone steps of 10 ms. Simulated BSP are displayed for the standard 12-lead ECG. The colors in the ECG correspond to the colors stated in the left column with the simulation characteristics. One cube in the 12-lead ECG corresponds to 40 ms (width) and 0.5 mV (height).

Hundred-fifty-three patch activation sequences were simulated for each of the different types; delayed (Figure 4), dyssynchronous (Figure 5) and discontinuous (Figure 6) with the same underlying normal ventricular activation sequence. By only slowing down the propagation velocity over and through the patch (Figure 4), the overall activation wave remains similar to normal ventricular activation, while the activation timing range over the patch increases. In case of dyssynchronous patch activation (Figure 5), the total patch activation timing increased and the effect of an inhomogeneous substrate is observed by increasing local activation timing differences. Discontinuous patch activation due to an ‘isolating’ layer between patch and normal ventricular myocardium, resulted in a patch activation initiated at the edge of the patch (Figure 6). The direction of activation through the patch differed depending on the connection between the ventricular myocardium and the patch. Abnormal patch activation affects both QRS morphology (fragmentation, amplitude changes) and/or QRS duration. Median (range) total activation duration for the simulation of the activation sequence was 114 (88; 279) ms with the RV free wall patch, 117 (88; 269) ms with the LV free wall patch, 114 (88; 211) ms with the RV tricuspid valve patch and 130 (88; 273) ms with the RVOT patch. For the severely prolonged activation sequences, observed amplitude at end-QRS was low. Overall QRS-CC was 0.99 (0.98; 1.00), and a significant difference (*p* < 0.001) between delayed *versus* dyssynchronous and continuous *versus* discontinuous patch activation was observed (Table 2). Compared to dyssynchronous patch activation (Figure 5), homogeneous delayed patch activation (Figure 4) showed limited effect on the simulated BSPs. The effect of the different types of discontinuous patch activation resulted in variable effect within the QRS complex when assessing the onset and duration of abnormal BSP (Figure 6). When comparing discontinuous type 1 to type 2, the effect on QRS morphology is less for type 1. In type 2, there are clear signs of late activation in the simulated BSP after complete ventricular activation. In epicardial vs. endocardial disease (Figure 7), the effect on the BSP of the abnormal activation sequence is opposite. Heuristically decreasing source strength of the patches resulted in a decrease in amplitude of the potentials generated by the patch on the BSP (Figure 8). The lead position in which the BSP waveform is affected most is directly related to the location of the diseased myocardium (Figure 9A). Furthermore, the vicinity of the patch to a site of early ventricular activation is related to the timing of initial QRS morphology changes (Figure 9B).

**TABLE 2:** Overview of simulation characteristics and resulting QRS-CC.

Patch simulation type	Median	IQR1	IQR3	Min	Max
Delayed patch activation	1.00	0.99	1.00	0.20	1.00
Delayed type 1 patch activation	1.00	1.00	1.00	0.96	1.00
Delayed type 2 patch activation	1.00	1.00	1.00	0.96	1.00
Dyssynchronous patch activation	0.97	0.92	0.99	−0.28	1.00
Dyssynchronous type 1 patch activation	0.98	0.93	1.00	−0.09	1.00
Dyssynchronous type 2 patch activation	0.98	0.95	1.00	0.12	1.00

### 3.2 Clinical case—Invasive electro-anatomical mapping

**FIGURE 10:**
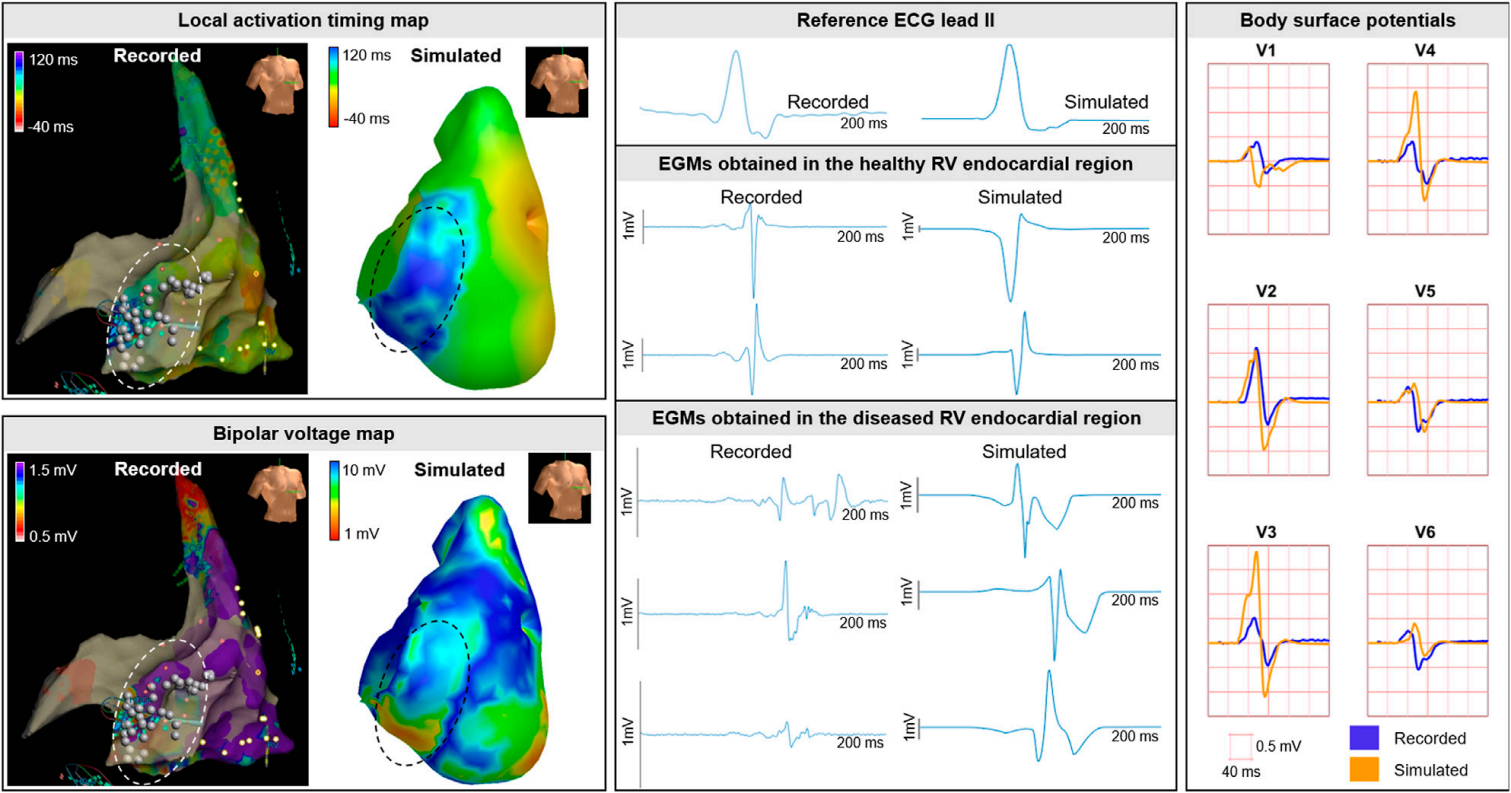
Clinical EAM case. Recorded and simulated electro-anatomical mapping (EAM). Local activation timing and bipolar voltage maps of the RV endocardium are displayed from red (early/low) to blue (late/high). Recorded and simulated bipolar cardiac electrograms are displayed for healthy and diseased regions. Within diseased regions (dashed circles), fragmentation and late potentials occur in recorded and simulated electrograms. Ablation was performed and indicated with the white dots. Recorded and simulated body surface potentials. The BSP is displayed per lead for the QRS complex for the precordial signals V1-V6 from the standard 12-lead ECG. One cube in the 12-lead ECG corresponds to 40 ms (width) and 0.5 mV (height).

Invasive recorded and simulated local activation timing maps and bipolar voltage maps were created (Figure 10). For the recorded and simulated EAM, spacing between unipolar electrograms was 3 mm and 5 ± 1 mm, respectively. The recorded and simulated local activation times ranged between 0–130 and 0–132 ms, respectively. All electrograms considered were obtained prior to ablation. Median bipolar voltage of these EAM electrograms was 0.5 *versus* 4.5 mV in the regions within and outside the diseased area. For the simulated maps, median bipolar values were 7.1 and 11.8 mV within and outside the simulated diseased area, respectively. In both the recorded and simulated cardiac electrograms, fractionation and late potentials were observed in the diseased areas (Figure 10).

In the included patient (QRS duration 100 ms) who underwent EAM, fragmentation and low-QRS amplitude was observed in the recorded BSP in leads located at the right anterior side of the chest. The morphology of the QRS complexes comparing recorded to simulated (Figure 10), were similar and fragmentation at end-QRS was observed.

## 4 Discussion

In this article we introduce a new method to simulate local myocardial disease and its effect on simulated EDL-based potentials. This new method enables the simulation of activation sequences in myocardium with strands/islands of surviving and fibrofatty tissue. Different types of myocardial disease with specific activation characteristics (delay, dyssynchrony, and discontinuity) can be simulated which directly gives insight into the effect on simulated BSP which can in turn be related to different cardiac diseases. The findings from the simulation study were substantiated by the invasive EAM case of a patient with arrhythmogenic cardiomyopathy. The incorporation of structural defects with partially surviving myocardium in EDL-based ECG simulation is relatively easy to implement. The technique not only can aid teaching in the context of ECG waveform changes due to specific pathology, but might also be used in the non-invasive inverse estimation of activation in the presence of inhomogeneous scar, as occurs in for example arrhythmogenic cardiomyopathy or Brugada syndrome.

### 4.1 Effect of structural abnormalities on potentials

This new EDL method increases the understanding of ECG waveform changes due to diseased myocardium with distinct activation sequences. By changing the simulation characteristics like propagation velocity, activation wave dyssynchrony/discontinuity and source strength, the effect of abnormal patch activation can be investigated and related to waveform changes in both electrograms and BSP. With the described method, the general effect of a substrate consisting of both fibrofatty and healthy myocardium is modeled, thereby capturing the overall effect of a certain substrate on observed potentials. The patch may also be used to represent scar with small channels of viable tissue, which may provide isthmuses for reentry. By adjusting the connection between patch and ventricular tissue, such a scar can be modeled, providing also the possibility for a mid-myocardial point of reentry.

In regions with severe scarring, low-voltage, late and fragmented potentials are observed during EAM procedures (De Bakker et al., 1993; Marchlinski et al., 2000; Soejima et al., 2002; Aliot et al., 2009; Glashan et al., 2018) as well as in our simulations (Figure 10). However, the presence of myocardial disease cannot always be identified in recorded BSP, possibly due to the anatomical location of the substrate, its vicinity to an early site of activation (Figure 5) or due to the low local changes in potentials (Klein et al., 1982; De Bakker et al., 1983), resulting in relatively small, possibly mid-QRS changes (Prineas et al., 2009). Whereas such pathological changes may not be as apparent as fragmentation (Das and Zipes, 2009) or prolonged terminal activation duration (Cox et al., 2008), they still may be highly relevant to monitor disease progression. The presence of scarred tissue can be assessed using late gadolinium enhancement (LGE)-CMR imaging (Iles et al., 2015). Regions with LGE are associated with the presence of abnormal electrocardiograms during EAM and ventricular arrhythmias (Di Marco et al., 2017). By also taking into account areas with LGE presence, patient specific EDL-based modeling and risk-stratification may be further improved.

### 4.2 Clinical implications to monitor disease progression

In this study, we observed that subtle changes in the cardiac activation were not always visible in the standard 12-lead ECG (Figure 5). In inherited cardiomyopathies, subtle changes in cardiac activation may however be a sign of disease progression and increased risk for ventricular arrhythmias. For instance; in arrhythmogenic cardiomyopathy, most structural and electrical signs of myocardial disease are observed in the basal area of the RV free wall (Corrado et al., 1997; Marcus et al., 2010; Te Riele et al., 2013). When modeling this type of disease, the largest changes in BSP occurred in leads not included in the standard 12-lead ECG (Figure 5). A previous study using echocardiography (Mast et al., 2019) observed early structural signs of disease in the absence of ECG abnormalities in the 12-lead ECG. This may be explained by the fact that the standard 12-lead ECG inadequately images (subtle) ECG changes due to substrate location. Furthermore, the current task force criteria for arrhythmogenic cardiomyopathy mainly focuses on end-QRS ECG abnormalities (Marcus et al., 2010). However, depending on the location of the substrate and its vicinity to early sites of activation, changes throughout the QRS complex (e.g., changing RS-amplitude ratio) may be an important indicator of disease progression.

### 4.3 Identification of new ECG features to identify disease

The importance of the identification of subtle ECG changes to detect disease is also demonstrated within the field of ECG-based artificial intelligence. With deep neural networks, low ejection fraction (Yao et al., 2021), LV hypertrophy (Ko et al., 2020), early signs of inherited cardiomyopathy (van de Leur et al., 2021) and electrolyte imbalance (Galloway et al., 2019; Pilia et al., 2020) can be identified from the apparently normal 12-lead ECG. Furthermore, atrial fibrillation (Attia et al., 2019; Nagel et al., 2021) and life-threatening ventricular arrhythmias (Ko et al., 2020) can be predicted using the 12-lead ECG during sinus rhythm. The findings of ECG-based deep neural networks may be further substantiated through ECG simulation. By focusing on the underlying pathology, observed pathological waveform changes in the 12-lead ECG may be explained by systematically evaluating the effect of substrates on QRS morphology. Thus, together with ECG-based artificial intelligence, the new ECG simulation technique may aid the discovery of yet unidentified pathological waveform changes to detect and monitor disease onset and progression.

### 4.4 Patch simulation characteristics

To demonstrate the effect of different types of patch activations on BSP, patch source strength was set equal to ventricular source strength and propagation velocities to simulate delayed activation were set far below (0.25 m/s) real physiological values (0.85 m/s). With the simulation of an isolating mid-myocardial layer (discontinuous activation type 1&2), a mid-myocardial line of block was simulated and activation sequences and BSPs were both clearly affected (Figure 6). Without this isolating layer, changes in BSP were observed in the initial part of the QRS complex. With an isolating layer, the type of connection determined the effect on simulated BSP; for type 1 disconnection, the effect on BSP was difficult to distinguish from normal, whereas the effect of type 2 was clearly observed in the ST-segment as fragmentation.

Whereas simulated propagation velocity values, the homogeneous isolating mid-myocardial layer and used source strength were not completely realistic for in-human substrates, the effect of abnormal activation sequences on simulated BSP was clearly distinguishable. In reality, source strength of diseased myocardium is lower compared to healthy myocardium due to the presence of fibrofatty tissue (Klein et al., 1982; Glashan et al., 2018). By decreasing patch source strength, the presence of fibrofatty tissue was accounted for, directly resulting in less evident effect on the BSP (Figure 8). This finding is in line with observations during clinical EAM; where fragmented and late potentials can be observed without evident pathological signs in recorded BSP.

### 4.5 Comparison to other EDL-models of disease

The fundamental difference between our new method and the method to simulate transmural scar by creating a hole in the ventricular anatomical model, is the ability to model the presence of partially-*versus* completely electrically inactive myocardium (Oostendorp and Ham, 2001). While creating a hole in the segmented ventricular anatomical model is appropriate to simulate a region, that is, completely electrically inactive, it is not for partially active substrates (Klein et al., 1982; De Bakker et al., 1983; Aliot et al., 2009; Glashan et al., 2018). With patches, the presence of diseased and healthy myocardium within the same region was modeled, similarly to incorporating a fibroblast model. This method thus serves as a more adequate representation of myocardium in e.g., border zones of old myocardial infarctions or substrates in inherited cardiomyopathies. In the current study we embedded epicardial and endocardial patches, which cannot represent transmural scar. In future studies, we aim to combine both methods by creating a hole and filling this hole with a patch representing locally transmurally diseased tissue.

### 4.6 Enhancing our understanding of pathological BSP

Nowadays, thorough understanding of the relation between pathological cardiac electric activity and corresponding BSP requires extensive electrophysiological and anatomical training within the field of electrophysiology. However, for teaching purposes, a tool to demonstrate this relation provides important insight in the basic aspects of electrocardiography. By interactively testing the effect of different disease-types on the activation sequence in the well-known known *ECGsim*-tool (van Dam et al., 2010; van Oosterom et al., 2011), their effect on corresponding BSP can be directly observed and teaching of the aspects of electrocardiography is further improved. Furthermore, from insights obtained from ECG simulations, non-invasive inverse estimation of activation sequences can be further optimized to further improve early detection and disease risk-stratification.

### 4.7 Limitations

In this study, we used a ventricular activation sequence initiated at six different sites with an average myocardial conduction velocity of 0.85 m/s as our normal reference to study the effect of different patch activation on the QRS wave. We are aware that this ventricular activation sequence resulted in ECG patterns clinically categorized as abnormal (*e.g.,* V1 RS pattern, prominent S in V6, aVR QS pattern, and aVL QR pattern). These observations are likely to be the consequence of the relatively simple representation of normal ventricular activation, compared to complex true activation sequence. As the main goal of this study is to demonstrate the *change* by the presence of scar tissue, this does not compromise the conclusions of the study. The new method described in this article was not strictly validated in the current study. The comparison shown in Figures 3–8 and the comparison with clinical data (Figure 10) give strong evidence, that the new method produces reasonable results. Future studies will focus on testing the validity of this new EDL approach for modeling different cardiac diseases. Depending on the type of substrate and disease severity, the most appropriate method to model the local activation wavefront can be chosen.

When embedding a patch in the ventricular model a local dent was created consequently affecting the ventricular distance matrix. As activation sequences were computed using the fastest route algorithm, the local dent directly affected computed activation sequences and corresponding BSP. In tissue surrounding diseased areas, a decrease in activation times is not expected as anisotropy is likely to more pronounced in this tissue beneath the patches (*e.g.,* diseased tissue). We therefore assumed that when embedding a patch, waveform propagation locally slows in ventricular tissue. To model this, the distance matrix computed for a model without patches was used to compute ventricular activation sequences also in the ventricular model with embedded patches. From a physiological point of view, we effectively increased local anisotropy.

### 4.8 Conclusion

A new method to describe the effect of (partially) electrically active substrate in EDL-based ECG simulation was established. Changes in cardiac activation sequence were directly related to changes in BSP. Insights obtained from the simulation study were in agreement with the presented clinical cases. With this new method, further in-depth understanding of the effect of pathological activation sequences on BSP can be obtained. The method will be incorporated into the next-generation of ECGsim and thus be available to everybody. With these insights, risk-stratification and understanding of disease progression in cardiomyopathies may be further improved.

## Data Availability

The raw data supporting the conclusion of this article will be made available by the authors upon request with the corresponding author.
